# A Structured Review of Deep Learning Approaches and Image-Preprocessing Techniques for Automated Contact Allergy Patch Test Interpretation

**DOI:** 10.3390/medsci14020322

**Published:** 2026-06-15

**Authors:** Dominyka Stragyte, Gvidas Mikalauskas, Katrina Gaidulevic, Renata Paukstaitiene, Kestutis Stasaitis, Vidas Raudonis, Skaidra Valiukeviciene

**Affiliations:** 1 Faculty of Medicine, Medical Academy, Lithuanian University of Health Sciences, LT-44307 Kaunas, Lithuania; skaidra.valiukeviciene@lsmu.lt; 2 Department of Skin and Venereal Diseases, Lithuanian University of Health Sciences, LT-44307 Kaunas, Lithuania; gvidas.mikalauskas@stud.lsmu.lt (G.M.); katrina.gaidulevic@stud.lsmu.lt (K.G.); 3 Department of Physics, Mathematics and Biophysics, Lithuanian University of Health Sciences, LT-44307 Kaunas, Lithuania; renata.paukstaitiene@lsmu.lt; 4 Department of Emergency Medicine, Lithuanian University of Health Sciences, LT-44307 Kaunas, Lithuania; kestutis.stasaitis@lsmu.lt; 5 Automation Department, Faculty of Electrical and Electronics Engineering, Kaunas University of Technology, LT-51368 Kaunas, Lithuania; vidas.raudonis@ktu.lt

**Keywords:** patch test, allergic contact dermatitis, contact dermatitis, deep learning, convolutional neural network, artificial intelligence, machine learning

## Abstract

**Background**: Allergic contact dermatitis (ACD) is a common inflammatory skin disease and patch testing (PT) remains the gold standard for its diagnosis; however, PT interpretation is time-consuming and prone to inter-observer variability. Growing advances in digital imaging and artificial intelligence (AI) have encouraged the development of automated PT evaluation systems. This review aimed to summarize the use of deep learning networks (DNNs) and image-preprocessing techniques for PT classification. **Methods**: A literature review was conducted to identify original research published between 2020 and 2025 that applied deep learning algorithms to PT image analysis. Included studies were assessed with respect to model architecture, dataset characteristics, preprocessing strategies, and diagnostic performance. **Results**: Six original studies employing deep learning for PT image classification met the inclusion criteria. They employed a range of architectures, including YOLOv5x, EfficientNetB0, Xception, and custom CNN models. Reported diagnostic performance varied, with accuracy values ranging from 90% to 99.5%, F1-scores from 0.37 to 0.98, and AUROC values up to 0.94. Despite promising results, models remain unreliable for ICDRG grading, especially for severe reactions, and methodological variability in dataset composition, imaging conditions, preprocessing pipelines, and classification tasks limits comparability across studies. **Conclusions**: Deep learning shows promise for automated PT interpretation, but further standardized and multicenter studies with detailed preprocessing protocols and comprehensive ICDRG grading are required for clinical implementation.

## 1. Introduction

Allergic contact dermatitis (ACD) represents one of the most common inflammatory skin diseases encountered in dermatological practice, with prevalence estimates ranging from 15% to 20% in the general population [1,2]. ACD is most often triggered by various substances, including metals (particularly nickel and cobalt), fragrances, preservatives, rubber additives, and topical medications [3,4]. The gold standard for diagnosing ACD is the patch test (PT), standardized by the International Contact Dermatitis Research Group (ICDRG) [5]. PT interpretation varies between clinicians and requires time-consuming evaluation [6,7].

Digital imaging is increasingly implemented in clinical practice and PT documentation [8]. The digitalization of cutaneous pathology allows for the integration of artificial intelligence (AI) into medical data analysis [9]. Studies have shown that multilayer neural networks with manual feature engineering can achieve expert-level accuracy in detecting melanoma, basal cell carcinoma, and inflammatory diseases from images [10,11,12]. However, deep neural networks (DNNs), particularly convolutional neural networks (CNNs), automatically learn features from raw pixels and are regarded as a modern, accurate, and appropriate method for image interpretation [13,14].

DNNs are considered appropriate for the evaluation of PT, as ICDRG categories standardize the visual characteristics of allergic reactions, including erythema, papules, vesicles, and infiltration [15,16,17]. To our knowledge, only a limited number of studies have analyzed DNNs for PT interpretation, and these investigations differ substantially in methodology, algorithms, and statistical approaches [18,19,20,21]. Despite the widespread adoption of AI in dermatology, automated PT interpretation remains a relatively new and evolving field.

In recent years, increased accessibility of high-resolution digital imaging and the growing availability of annotated dermatological datasets have accelerated progress in AI-assisted diagnostic tools. These advances have led to the exploration of automated systems aimed at supporting clinicians in tasks traditionally dependent on subjective visual assessment. As PT reactions can present subtle gradations of erythema or minimal vesiculation, consistent evaluation requires both experience and careful inspection. AI-based systems could serve as decision-support tools that help standardize reaction classification, reduce observer-dependent variability, and potentially improve diagnostic accuracy. Furthermore, the rapid development of smartphone cameras and portable imaging devices has created new opportunities to capture PT images outside specialized clinical settings, increasing the feasibility of remote or teledermatology-based PT interpretation.

Nevertheless, the application of AI to PT analysis presents specific challenges. Patch test reactions often involve heterogeneous skin features influenced by patient skin phototype, anatomical site, lighting conditions, and temporal progression of allergic inflammation. These factors may complicate automated image interpretation and limit generalizability unless properly accounted for during model training. Moreover, the scarcity of severe reactions in clinical datasets introduces class imbalance—a major obstacle when developing DNN models capable of differentiating clinically important ICDRG grades. These considerations highlight the need for comprehensive evaluations of the strengths and limitations of AI-based PT classification.

This review presents a comprehensive overview of DNN applications for the interpretation of patch test reactions, emphasizing methodological trends, current limitations, and future directions for research in this emerging field.

## 2. Materials and Methods

### 2.1. Literature Search and Selection Strategy

We conducted a literature review following the Preferred Reporting Items for Systematic Reviews and Meta-Analyses (PRISMA) guidelines to identify and analyze studies employing DNNs for PT classification [22].

### 2.2. Database Selection

During the period from 1 November to 1 December 2025 four major databases were searched: PubMed (MEDLINE), Scopus, IEEE Xplore, and Google Scholar. PubMed provided comprehensive coverage of biomedical literature, Scopus offered broad scientific literature access, IEEE Xplore captured engineering and computer science publications, and Google Scholar served to identify grey literature and ensure comprehensive coverage.

### 2.3. Search Terms

The search strategy employed both Medical Subject Headings (MeSH) terms and free-text keywords combined with Boolean operators: (“patch test” OR “patch testing” OR “contact dermatitis” OR “allergic contact dermatitis” OR “epi-cutaneous testing”) AND (“deep learning” OR “convolutional neural network” OR “CNN” OR “artificial intelligence” OR “machine learning” OR “neural network” OR “computer vision”) AND (“classification” OR “detection” OR “diagnosis” OR “automated” OR “automatic” OR “recognition”).

### 2.4. Inclusion Criteria

1. Original research articles published in peer-reviewed journals or conferences.
2. Studies employing DNN methods (CNNs, recurrent networks, transformers, or hybrid architectures) for patch test analysis.
3. Publications in English.
4. Studies using digital images of PT reactions.
5. Publications from January 2020 to December 2025.

### 2.5. Exclusion Criteria

1. Review articles, systematic reviews, meta-analyses, editorials, or commentaries.
2. Conference abstracts without full-text availability.
3. Studies using non-DNN machine learning methods.
4. Studies focusing solely on other forms of allergy testing (e.g., skin prick tests, intradermal tests).
5. Duplicate publications of the same dataset.
6. Publications in languages other than English.

### 2.6. Search and Selection Process

**Figure 1:**
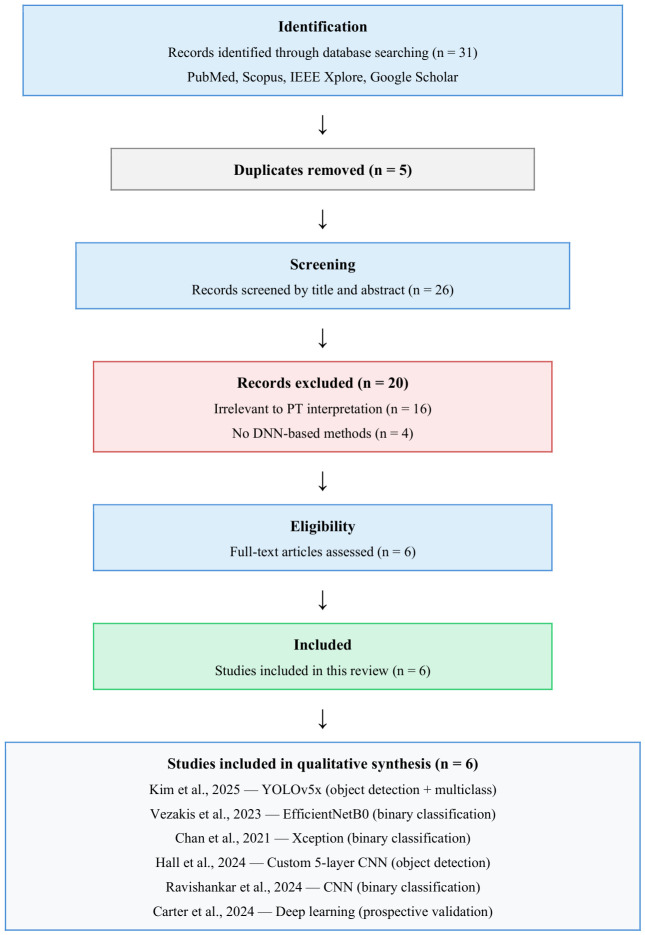
Literature search strategy for this review [18,19,20,21,23,24].

The database search identified 31 records. After removal of duplicate records (n = 5), a total of 26 unique records remained and were screened based on titles and abstracts. During this screening stage, 20 records were excluded for not meeting the inclusion criteria, primarily due to irrelevance to PT interpretation (n = 16) or the absence of DNN-based methods (n = 4). Six studies met the predefined inclusion criteria. The study selection process is summarized in the PRISMA flow diagram (Figure 1).

## 3. Results

### 3.1. Included Studies

Six studies met the predefined inclusion criteria and were included in the final analysis. All identified studies investigated artificial intelligence-based approaches for automated interpretation of patch test (PT) reactions using digital images of the skin. Four of the studies were conducted using prospective study designs, while two studies relied on retrospective clinical data collected over longer periods. Despite differences in study design, all studies shared a similar objective: to evaluate whether deep learning algorithms could assist in the interpretation of allergic skin reactions detected during patch testing.

The included studies demonstrated substantial methodological variability in dataset composition, imaging methods, preprocessing pipelines, and machine learning architectures. In particular, the size of the datasets differed considerably between studies. Kim et al. utilized the largest dataset, consisting of tens of thousands of images from more than three thousand patients [18]. In contrast, other studies relied on considerably smaller datasets, ranging from approximately several hundred to several thousand images [19,20,21].

Another important difference concerned the definition of the classification task. Two studies attempted to approximate the clinical interpretation process by incorporating the ICDRG grading system, which categorizes PT reactions according to severity [18]. In contrast, other studies simplified the classification task by performing binary classification of reactions as either positive or negative [19,20,21]. While binary classification may simplify model training and improve accuracy metrics, it does not fully reflect the complexity of clinical patch test interpretation.

The studies also differed in the level of automation of the analytical workflow. One study implemented a deep learning object detection framework capable of automatically locating PT sites within images [18]. In contrast, the remaining studies required manual identification or cropping of patch test regions prior to model analysis, which introduces additional manual effort and may limit scalability in routine clinical practice [19,20,21].

Despite these methodological differences, all six studies reported promising performance metrics for AI-based PT interpretation, with overall accuracy values ranging from approximately 90% to nearly 99.5% [18,19,20,21,23,24]. However, important limitations were also noted, particularly related to dataset imbalance and the limited representation of severe allergic reactions.

### 3.2. YOLOv5x-Based Object Detection

Kim et al. developed a deep learning model based on the YOLOv5x object detection architecture designed to automatically identify patch test sites and simultaneously classify reaction severity according to the ICDRG scoring system [18]. This approach represents one of the most advanced workflows among the analyzed studies because it integrates both object localization and classification within a single deep learning framework.

The dataset used in this study was the largest among all included studies, comprising 86,477 clinical images obtained from 3203 patients undergoing patch testing [18]. The images were acquired using high-resolution DSLR cameras under standardized clinical conditions, which allowed for consistent image quality and reduced variability in lighting and image resolution. The large dataset size provided the model with substantial training data and likely contributed to the high-performance metrics reported in this study.

However, a major limitation of the dataset was the extreme imbalance in reaction classes. More than 96% of the recorded PT reactions were negative, while positive reactions were relatively rare [18]. Furthermore, the dataset did not include any grade 3 or grade 4 reactions, which represent the most severe allergic responses according to the ICDRG classification system. This imbalance introduces potential bias in model training and may result in overly optimistic accuracy values driven by correct classification of the dominant negative class.

The YOLOv5x model demonstrated strong performance, achieving an accuracy of 0.983 at both 24 h and 48 h evaluation time points, an F1-score of 0.982, and a sensitivity of 0.997 for identifying negative reactions [18]. These results indicate that the model was highly effective in detecting non-reactive PT sites. However, due to the limited representation of positive and severe reactions, the model’s ability to reliably detect clinically significant allergic responses remains uncertain.

A key advantage of the YOLO-based approach is the elimination of manual segmentation steps. Traditional CNN-based pipelines typically require manual cropping of PT regions prior to analysis. By contrast, object detection frameworks allow the model to analyze the entire image and automatically identify relevant test sites. This capability represents a significant step toward the development of fully automated PT interpretation systems.

### 3.3. EfficientNetB0-Based Binary Classification

Vezakis et al. applied an EfficientNetB0 convolutional neural network to classify patch test reactions using standardized dermatological images [19]. EfficientNet architectures are designed to achieve strong classification performance while maintaining relatively efficient computational requirements, making them suitable for medical imaging applications.

The dataset consisted of 1579 images obtained from 200 patients undergoing patch testing [19]. Compared with the dataset used by Kim et al., this dataset was considerably smaller, which may limit the generalizability of the trained model. However, the images were acquired using the Antera 3D^®^ imaging system (Miravex Limited, Dublin, Ireland), which provides standardized illumination and imaging conditions. Such standardization may improve the quality and consistency of training data.

Unlike the YOLO-based approach, the EfficientNetB0 model required manual selection of regions of interest before classification. Researchers manually cropped PT sites from the original images, and these cropped regions were then used as input for the neural network [19]. While this preprocessing step simplifies the classification problem, it also introduces additional annotation work and reduces the level of automation.

To compensate for the relatively small dataset size, the authors applied data augmentation techniques, including horizontal and vertical flipping of images. Data augmentation increases dataset diversity and helps prevent overfitting during model training.

The model achieved approximately 90% accuracy, an F1-score of 0.83, recall above 86%, and specificity of 94% in identifying PT reactions [19]. These results suggest that the model could distinguish between reactive and non-reactive sites with reasonable reliability.

However, the study employed binary classification, grouping all positive reactions together regardless of severity. While this approach simplifies model training, it limits clinical interpretability because it does not differentiate between mild, moderate, and severe allergic reactions according to the ICDRG scale.

### 3.4. Xception-Based Smartphone Image Classification

Chan et al. investigated the potential of smartphone-based imaging combined with deep learning for automated interpretation of PT reactions [20]. In this study, an Xception convolutional neural network was used to classify cropped PT image regions.

The dataset included 77 patients and approximately 5900 images, which were obtained from standardized patch test panels [20]. Compared with other studies included in this review, the dataset was relatively small, particularly in terms of patient numbers. Nevertheless, the use of smartphone images introduces an important practical dimension, as smartphone-based imaging could facilitate remote or decentralized PT evaluation.

One of the primary challenges associated with smartphone photography is variability in image acquisition conditions, including lighting, camera settings, and image resolution. To address this issue, the authors implemented several image-preprocessing techniques, including normalization of color, brightness, and intensity [20]. These preprocessing steps were designed to reduce variability between images and ensure that the neural network focuses on clinically relevant features rather than imaging artifacts.

The Xception model performed binary classification of PT reactions, distinguishing between positive and negative responses. The reported results were highly promising, with 99.5% accuracy and an F1-score of 0.89 [20].

Despite the high reported accuracy, the relatively small dataset size raises concerns about potential overfitting and limited generalizability. Additionally, the study relied on cropped image regions, meaning that the model did not perform automated detection of PT sites. Therefore, manual preprocessing would still be required before classification.

Nevertheless, the study demonstrates the feasibility of using smartphone imaging in combination with deep learning models for automated PT interpretation.

### 3.5. Custom CNN with Class Rebalancing

Hall et al. developed a custom five-layer convolutional neural network designed to detect and classify PT reactions using a dataset collected over a ten-year period [21]. This study differed from the others in that it explicitly addressed the issue of class imbalance during model training.

The dataset included 201 patients and more than 2800 image tiles derived from PT images [21]. Because the dataset was collected retrospectively, it likely contained greater variability in imaging conditions compared with prospective studies.

To mitigate the dominance of negative reactions, the authors implemented class rebalancing techniques during model training. These methods aim to increase the importance of minority classes in the learning process.

Despite these efforts, the model achieved 90.9% accuracy, 70.1% sensitivity, 91.7% specificity, and an F1-score of 0.37 [21]. The relatively low F1-score indicates that the model struggled to reliably detect positive reactions.

This finding highlights the challenges associated with training deep learning models on highly imbalanced medical datasets. Even when class rebalancing techniques are applied, models may still struggle to accurately identify rare positive cases.

### 3.6. CNN-Based Binary Classification with Large Dataset

Ravishankar et al. conducted a retrospective analysis of patch test images collected between March 2020 and March 2021 to evaluate the performance of a convolutional neural network as a binary classifier for PT reaction detection [23]. The study included 13,622 images obtained from 125 patients, representing one of the larger datasets among the reviewed studies in terms of individual image count.

The CNN model was trained to discriminate between reaction and non-reaction patches using cropped images of individual allergen sites. The images were resized to 100 × 100 pixels and processed through a standard CNN architecture. The model achieved an area under the receiver operating characteristic curve (AUC) of 0.940 and an accuracy of 90.1%, indicating strong discriminative ability between reactive and non-reactive PT sites [23].

An important limitation of the study was the demographic composition of the patient cohort. The majority of patients were female (81.6%), and 88.0% had Fitzpatrick skin types I-II, which may limit the generalizability of the model to more diverse patient populations. The study employed binary classification, grouping all positive reactions regardless of severity, and required manual cropping of individual allergen sites prior to analysis.

Despite these limitations, the study provides additional evidence supporting the feasibility of CNN-based automated PT interpretation and demonstrates that reasonable classification performance can be achieved even with relatively simple CNN architectures when sufficient training data are available.

### 3.7. Prospective Clinical Validation Across Diverse Skin Types

Carter et al. conducted a single-arm prospective clinical trial to validate a deep learning algorithm for PT interpretation across all Fitzpatrick skin types [24]. This study represents the only prospective external validation study among the reviewed publications and is therefore of particular methodological significance.

A total of 206 evaluable participants were enrolled, with a mean age of 39 years. Sixty-six percent of participants were female, and notably, 47% had Fitzpatrick skin types IV–VI, ensuring representation of darker skin tones that are typically underrepresented in dermatological AI research [24]. Each participant was exposed to 10 allergens, and the reference standard was established by a board-certified dermatologist five days after initial patch placement. The deep learning algorithm processed photographs of the test sites obtained on the same day.

Forty-two percent of participants experienced one or more allergic reactions, resulting in a total of 132 allergic reactions across the cohort. The model demonstrated high discrimination with an AUROC of 0.86 (95% CI: 0.82–0.90) and specificity of 93% (95% CI: 92–94%), but achieved lower sensitivity of 58% (95% CI: 49–67%) [24]. Human expert readers evaluating the same photographs achieved comparable specificity but higher sensitivity, suggesting that the AI model was more conservative in identifying positive reactions.

This study is noteworthy for several reasons. First, it is the only prospective validation study in the field, providing a more rigorous assessment of real-world model performance compared with retrospective analyses. Second, the deliberate inclusion of diverse skin types addresses a critical gap in dermatological AI research, as most existing models are trained and validated predominantly on lighter skin types. Third, the comparison of AI performance with human expert readers provides important context for interpreting model metrics. The relatively low sensitivity highlights the ongoing challenge of reliably detecting positive reactions from photographs alone, particularly across diverse skin types.

### 3.8. Comparative Summary

Overall, the six included studies demonstrate the potential of deep learning methods for automated interpretation of patch test reactions (Table 1). Various CNN architectures were successfully applied to classify PT reactions with reported accuracy values ranging from approximately 90% to 99.5% and AUROC values up to 0.94 [18,19,20,21,23,24].

However, substantial methodological heterogeneity exists between studies, including differences in dataset size, imaging methods, preprocessing pipelines, and classification tasks. In particular, the majority of studies relied on binary classification, while only one study attempted multiclass classification according to the ICDRG severity scale [18].

Another important limitation across studies is dataset imbalance, with negative reactions greatly outnumbering positive reactions. This imbalance complicates model training and may lead to overly optimistic performance metrics.

Future research should focus on developing larger and more balanced datasets, improving automated detection of PT sites, and implementing standardized evaluation protocols to enable meaningful comparison of AI-based PT interpretation systems.

## 4. Discussion

Across all studies, conventional digital photography was used for image acquisition, although camera systems, image resolution, and lighting conditions varied considerably. Vezakis et al. applied the specific Antera 3D^®^ imaging system with artificial lighting, other studies utilized DSLR or smartphone cameras or selected one of these options. Patch testing protocols were based on established European or North American baseline PT series, and reaction grading followed ICDRG or closely related scoring systems. Kim et al. applied the ICDRG scoring system for grades 0–2, excluding grades 3 and 4 due to the absence of strong reactions. Other studies adopted a binary classification, considering grade 0 as negative and grades 1–4 as positive. All studies report high model performance, achieving accuracy (approximately 90–99.5%), strong F1-scores (up to ~0.98), however direct comparison is limited by differences in study design, tasks, and reported metrics.

Another important factor affecting the comparability of the analyzed studies is the variability in imaging conditions and standardization procedures. In dermatological image analysis, small differences in illumination, camera distance, or skin positioning can significantly affect the visual appearance of erythema, infiltration, and other reaction characteristics. Such variability may influence how deep learning models interpret visual features associated with allergic reactions. Standardized imaging systems, such as the Antera 3D^®^ device used by Vezakis et al., may therefore offer advantages in terms of reproducibility and consistent image quality. However, these systems may not always be available in routine clinical practice, where conventional digital cameras or smartphone photography are more commonly used. Consequently, the generalizability of AI-based PT interpretation systems may depend strongly on their ability to perform reliably across different imaging environments.

Despite these similarities, substantial heterogeneity in AI architectures, augmentation and detection types of positive PT sites limited comparison. The reviewed literature highlights variability in site detection approaches: automatic detection reported in only one Kim et al. research, manual segmentation in Vezakis et al., Hall et al., and Ravishankar et al., while Chan et al. and Carter et al. did not assess reaction localization. AI-based PT interpretation employed different DNN architectures, including five CNN models (EfficientNetB0, Xception, ResNet-based, a custom five-layer network, and a standard CNN classifier) and one YOLOv5x model (Kim et al.). Among the analyzed studies, the YOLOv5x algorithm involved the largest number of patients and images and developed automatic detection of PT areas as a key strength of research. However, the dataset was highly imbalanced, with 96% negative reactions, making it difficult to assess how accurately this model could identify positive reactions [18].

In addition to dataset imbalance, another challenge associated with AI-based PT interpretation is the limited availability of annotated clinical images representing different reaction severities. Severe allergic reactions are relatively rare in clinical practice, which naturally results in fewer examples available for model training. As a consequence, many datasets used in AI studies are dominated by negative or mild reactions, which may bias model learning. When models are trained predominantly on negative samples, they may achieve high overall accuracy but still fail to correctly identify clinically significant positive reactions. This phenomenon highlights the importance of using balanced datasets or advanced training techniques specifically designed to address class imbalance.

In contrast, the custom five-layer (CNN) model clearly applied class balancing techniques during training to reduce the dominance of negative reactions but required time consuming manual segmentation of PT areas [21]. EfficientNetB0 (CNN) also required substantial manual detecting of PT sites. Algorithm trained with the smallest dataset. To compensate, standard data augmentation techniques were applied to increase the diversity of training examples [19]. Xception (CNN) model lacked localization detection capability for positive PT areas but prioritized binary (positive and negative) PT reaction classification and preprocessing strategies, including normalization of color, brightness, and intensity [20].

The role of preprocessing techniques should also be emphasized when evaluating the performance of AI-based PT interpretation systems. Image-preprocessing methods, such as color normalization, contrast enhancement, and brightness adjustment, may significantly improve the ability of neural networks to detect subtle skin changes associated with allergic reactions. In particular, erythema and mild inflammatory responses can be difficult to distinguish under inconsistent lighting conditions. By standardizing image characteristics before model training, preprocessing techniques may reduce noise and improve the detection of clinically relevant features. Nevertheless, the optimal preprocessing pipeline for PT image analysis remains unclear, as different studies applied different techniques and reported varying results.

During this literature review, several unresolved methodological limitations on AI-based patch test analysis were identified. There are still no clear recommendations on the most appropriate methods for training AI systems, the choice of equipment used to obtain images, or the most effective strategies for preprocessing and data augmentation. First, most studies relied on small datasets, which limits the reliable evaluation and comparison of model performance. Second, heterogeneous AI architectures and training strategies impair objective comparison and identification of superior model. Third, there are still no clear recommendations regarding optimal imaging equipment, preprocessing and data augmentation techniques. Fourth, AI models still require substantial manual intervention, such as manual segmentation or compensation for data imbalance, which contradicts the fundamental aim of AI to reduce the workload of dermatologists.

Another important limitation concerns the lack of external validation across independent datasets. Many studies evaluate model performance using internal validation datasets derived from the same patient population used for training. Although this approach provides useful initial results, it does not guarantee that the model will perform equally well when applied to data from other institutions or imaging environments. Multicenter datasets and external validation studies are therefore essential for establishing the clinical reliability of AI-based PT interpretation systems.

Finally, the ICDRG scale is an appropriate standard; nevertheless, current models have a limited ability to accurately classify PT reactions according to ICDRG grades, particularly severe responses. Future studies should focus on larger, more balanced datasets and standardized methodologies to support reliable clinical implementation of AI-based patch test analysis and its diagnostic benefits. Furthermore, integration of automated detection algorithms with clinically interpretable grading systems may improve the usability of AI tools in dermatology and facilitate their adoption in routine clinical practice.

### 4.1. Situating Patch Test AI Within the Broader Landscape of AI in Dermatology

To contextualize the current state of AI-based PT interpretation, it is instructive to compare it with more established applications of deep learning in dermatology. AI-assisted melanoma detection, for instance, has benefited from over a decade of research, large publicly available datasets such as the ISIC archive containing hundreds of thousands of dermoscopic images, and multiple prospective validation studies demonstrating performance comparable to board-certified dermatologists [10,11]. Similarly, AI models for other skin conditions such as basal cell carcinoma and psoriasis have reached a level of maturity that permits consideration for clinical integration. By contrast, AI for PT interpretation remains in an early exploratory phase, characterized by small single-center datasets, a limited number of published studies, and no publicly available benchmark datasets. This comparison underscores both the potential and the distance yet to be covered before AI-based PT systems can approach the reliability and clinical readiness achieved in other dermatological domains.

### 4.2. Detection-Based Versus Classification-Based Approaches

An important methodological distinction among the reviewed studies concerns the choice between object detection and image classification frameworks. Detection-based approaches, such as the YOLOv5x model employed by Kim et al. [18], perform simultaneous localization and classification of PT reaction sites within full-panel images, thereby reducing the need for manual cropping and enabling end-to-end automation. Classification-based approaches, as implemented by Vezakis et al. [19], Chan et al. [20], and Hall et al. [21], require prior manual identification and segmentation of individual PT sites before classification. While classification-based models may achieve high per-patch accuracy, they introduce a dependency on manual preprocessing that limits scalability and clinical throughput. Detection-based models offer a more practical pathway toward fully automated PT interpretation but require substantially larger annotated datasets with bounding box labels. The choice between these paradigms has direct implications for clinical workflow integration and should be carefully considered in future study designs.

### 4.3. Performance Metrics: Beyond Overall Accuracy

A critical limitation observed across the reviewed studies is the reliance on overall accuracy as the primary performance metric. In the context of PT classification, where negative reactions constitute the overwhelming majority of cases, accuracy alone is insufficient and potentially misleading. A model that classifies all reactions as negative could still achieve accuracy exceeding 90% in datasets where 96% of reactions are negative, as observed in the dataset used by Kim et al. [18]. More informative metrics for clinically imbalanced datasets include sensitivity (the ability to correctly identify positive reactions), the F1-score (which balances precision and recall), and the area under the receiver operating characteristic curve (AUROC). The disparity between the high accuracy (90.9%) and low F1-score (0.37) reported by Hall et al. [21] clearly illustrates this issue: despite reasonable overall accuracy, the model failed to reliably detect positive reactions. Future studies should report a comprehensive set of performance metrics, including per-class sensitivity, specificity, positive predictive value, negative predictive value, and confusion matrices, to enable meaningful assessment of clinical utility.

### 4.4. The Gap Between Binary Classification and ICDRG Grading

The majority of the reviewed studies employed binary classification (positive vs. negative), which represents a substantial simplification relative to the ICDRG grading system used in clinical practice. The ICDRG system distinguishes between negative reactions, doubtful reactions, weak positive (+), strong positive (++), and extreme positive (+++) reactions. Clinically, the distinction between doubtful and weak positive reactions is particularly challenging and accounts for a significant proportion of inter-observer disagreement among dermatologists. For AI-based systems to provide clinically meaningful support, they must eventually progress beyond binary classification to address the full spectrum of ICDRG grades. However, this transition is complicated by the scarcity of severe reactions in available datasets and the inherent difficulty of distinguishing subtle gradations of erythema and infiltration from digital images alone. Bridging this gap will require both larger datasets enriched with rare reaction types and the development of models specifically designed for ordinal or multi-class classification of reaction severity.

### 4.5. Preprocessing Synthesis and Recommendations

Image preprocessing emerged as an important variable influencing model performance across the reviewed studies, yet no consensus exists regarding optimal preprocessing pipelines. Chan et al. [20] implemented the most comprehensive preprocessing approach, including color normalization, brightness adjustment, and intensity standardization, and reported the highest overall accuracy (99.5%). Vezakis et al. [19] benefited from the inherently standardized imaging provided by the Antera 3D system, which reduces the need for extensive post-acquisition preprocessing. Kim et al. [18] relied primarily on the consistency of DSLR imaging under controlled clinical conditions, while Hall et al. [21] dealt with heterogeneous imaging conditions from a retrospective dataset without detailed preprocessing. Based on the patterns observed across these studies, color normalization and brightness standardization appear to be particularly important when images are acquired using consumer-grade or smartphone cameras, where variability in lighting and white balance is greatest. For standardized clinical imaging systems, minimal preprocessing may suffice. Future studies should systematically evaluate the impact of individual preprocessing steps on model performance to establish evidence-based preprocessing recommendations. A more granular analysis of the preprocessing pipelines reveals that color normalization techniques varied across studies: Chan et al. employed histogram equalization combined with white balance correction to compensate for variable smartphone lighting; Vezakis et al. relied primarily on resizing and standard intensity normalization, as the Antera 3D system provides hardware-calibrated illumination; Kim et al. applied image resizing and YOLO-native augmentation (mosaic, mixup, random affine transformations); and Hall et al. used minimal preprocessing beyond cropping and resizing, which may partly explain the lower F1-score observed. The newly included Ravishankar et al. resized all images to 100 × 100 pixels and applied standard intensity normalization, while Carter et al. did not disclose preprocessing details in the available publication. Image resolution after preprocessing varied dramatically (from 100 × 100 pixels in Ravishankar et al. to 640 × 640 in YOLO-based pipelines), and this resolution difference likely affects the model’s ability to capture subtle features such as faint erythema borders, papular texture, and small vesicles. Based on these observations, we recommend that future PT image-preprocessing pipelines incorporate at minimum: (a) color normalization using a calibration reference (e.g., color checker chart visible in the image); (b) standardized minimum resolution of 224 × 224 pixels per individual allergen site; (c) reproducible cropping methodology with documented bounding box coordinates; and (d) data augmentation strategies appropriate to the dermatological domain (rotation, mild color jitter), avoiding aggressive augmentations that may distort reaction morphology.

### 4.6. Generalizability, External Validation, and Domain Variability

None of the reviewed studies performed external validation on independent datasets from different institutions. All models were evaluated using internal validation strategies, typically based on train-test splits from a single patient population. This approach limits confidence in the generalizability of reported performance metrics. Important sources of domain variability in PT imaging include differences in camera systems and image resolution, lighting conditions (natural vs. artificial, diffuse vs. directional), patient skin phototype (Fitzpatrick types I-VI), anatomical positioning and camera distance, and temporal variation in reaction progression. A recent prospective validation study by Carter et al. [24] evaluated a deep learning model for PT interpretation across all Fitzpatrick skin types and reported an AUROC of 0.86 with 93% specificity but only 58% sensitivity, highlighting the challenge of maintaining sensitivity in diverse clinical populations. Cross-institutional validation studies using standardized imaging protocols and diverse patient populations are essential for establishing the real-world reliability of AI-based PT systems. The development of shared benchmark datasets with consistent annotation standards would substantially accelerate progress in this area.

### 4.7. The Role of Transformer-Based Architectures

All six reviewed studies employed convolutional neural network architectures, which represent the dominant paradigm in medical image classification. However, recent advances in computer vision have demonstrated the potential of Vision Transformer (ViT) architectures [25], which use self-attention mechanisms to model long-range dependencies in images. Transformer-based models have shown competitive or superior performance compared with CNNs in several medical imaging tasks, including skin lesion classification and histopathological analysis. To date, no published study has applied transformer-based architectures to PT image classification. Given that PT reactions involve subtle textural and color changes that may benefit from the global contextual understanding offered by self-attention mechanisms, transformer-based approaches represent a promising direction for future research. Hybrid architectures combining CNN feature extraction with transformer-based classification heads may also offer advantages in this domain.

### 4.8. Transfer Learning: Opportunities and Limitations

All reviewed studies that employed established CNN architectures relied on transfer learning from models pretrained on the ImageNet dataset, a large-scale collection of natural images spanning 1000 object categories. Transfer learning has become a standard practice in medical image analysis, enabling models to leverage general visual features (edges, textures, shapes) learned from millions of natural images and adapt them to domain-specific tasks with limited training data. In the context of PT classification, EfficientNetB0 (Vezakis et al. [19]) and Xception (Chan et al. [20]) both utilized ImageNet-pretrained weights as initialization, while Kim et al. [18] employed a pretrained YOLOv5x backbone.

However, the suitability of ImageNet-derived features for PT image analysis warrants critical examination. PT reactions are characterized by subtle dermatological features including gradations of erythema, papular infiltration, vesiculation, and bullous formation, which differ substantially from the object categories represented in ImageNet. The low-level features (edges, color gradients) learned from natural images transfer well across domains, but mid-level and high-level features may be less relevant for distinguishing between, for example, a doubtful reaction and a weak positive reaction. Studies in other medical imaging domains have demonstrated that domain-specific pretraining (e.g., on dermatological image collections) can outperform ImageNet pretraining, particularly for tasks requiring fine-grained discrimination of subtle clinical features. Future PT classification studies should consider pretraining on large dermatological image datasets, such as the ISIC archive, which may provide more relevant intermediate feature representations. Additionally, self-supervised pretraining approaches, which do not require labeled data, represent a promising alternative for leveraging unlabeled PT image collections.

### 4.9. Temporal Variability in Patch Test Reactions

An important clinical consideration that has received insufficient attention in the reviewed studies is the temporal dynamics of PT reactions. Standard clinical protocols require readings at multiple time points, typically at 48 h (when patches are removed) and again at 72 or 96 h. Reactions may evolve significantly between readings: irritant reactions tend to diminish over time, while true allergic reactions typically intensify or persist. The ICDRG grading system is applied at each time point, and discordant readings between time points are clinically informative for distinguishing allergic from irritant responses.

Among the reviewed studies, Kim et al. [18] captured images at both 24 and 48 h, Vezakis et al. [19] at 48 and 72 h, Chan et al. [20] at day 4 only, and Carter et al. [24] at day 5. Hall et al. [21] and Ravishankar et al. [23] included images from multiple time points without explicitly modeling temporal progression. No study incorporated temporal information as a feature for classification, meaning that the dynamic evolution of reactions was not leveraged by any model. This represents a significant missed opportunity, as the temporal trajectory of a reaction contains clinically valuable information that could improve classification accuracy and, critically, help distinguish allergic from irritant contact dermatitis. Future studies should explore architectures capable of integrating multi-timepoint information, such as recurrent neural networks, temporal convolutional networks, or attention mechanisms applied across sequential images of the same test site.

### 4.10. Why Certain Architectures Perform Differently: A Technical Perspective

The substantial variation in reported performance across the reviewed studies reflects not only differences in dataset size and composition but also fundamental architectural distinctions that merit technical analysis. The highest accuracy (99.5%) was reported by Chan et al. [20] using Xception, a CNN architecture employing depthwise separable convolutions that efficiently capture spatial hierarchies while reducing parameter count. This strong performance likely reflects both the comprehensive preprocessing pipeline (color normalization, brightness adjustment) and the relatively controlled imaging conditions using smartphone photography with standardized protocols. However, it should be noted that this study evaluated only 77 patients, and the high accuracy may partly reflect limited dataset diversity.

By contrast, Kim et al. [18] achieved 98.3% accuracy with YOLOv5x on the largest dataset (86,477 images from 3203 patients). YOLOv5x employs a feature pyramid network with cross-stage partial connections, enabling multi-scale detection of reactions at various sizes within full-panel images. The detection-based approach eliminates the need for manual cropping and can simultaneously localize and classify multiple reaction sites, offering a practical advantage for high-throughput clinical settings. The lower performance of Hall et al. [21] (F1 = 0.37 despite 90.9% accuracy) likely reflects the combination of a simple five-layer custom CNN architecture with a heterogeneous retrospective dataset and pronounced class imbalance, demonstrating that architectural simplicity becomes a liability when imaging conditions and class distributions are challenging. The relatively simple CNN used by Ravishankar et al. [23] achieved AUC = 0.940, suggesting that with sufficient data volume (13,622 images), even architecturally simpler models can achieve reasonable discrimination, though the study population was demographically limited (88% Fitzpatrick types I–II).

### 4.11. Quantitative Synthesis and Limitations of Statistical Comparison

A formal quantitative meta-analysis of the six included studies was not feasible due to substantial heterogeneity in study design, dataset composition, evaluation methodology, and reported metrics. The included studies differ in patient populations (ranging from 77 to 3203 patients), dataset sizes (1579 to 86,477 images), imaging modalities (DSLR, smartphone, Antera 3D system), classification tasks (binary vs. multiclass; classification vs. object detection), and reference standards. Critically, only four of the six studies report directly comparable accuracy values, and only three report F1-scores; AUROC is reported in only three studies, and per-class sensitivity and specificity are reported inconsistently. Under these conditions, applying pooled meta-analytic techniques such as random-effects models or summary receiver operating characteristic (sROC) curves would produce statistically unreliable estimates and potentially misleading conclusions.

Despite the absence of formal pooling, several quantitative patterns are evident from cross-study comparison. Reported accuracy values cluster in two distinct ranges: studies employing comprehensive preprocessing and controlled imaging (Chan et al. 99.5%, Kim et al. 98.3%) achieved markedly higher accuracy than studies relying on heterogeneous retrospective imaging without standardized preprocessing (Hall et al. 90.9%, Ravishankar et al. 90.1%). The F1-scores show a substantially wider range (0.37 to 0.98), reflecting the impact of class imbalance and model sensitivity differences more sensitively than accuracy. The mean reported accuracy across the six studies is 94.5% (standard deviation 4.4%), while the mean F1-score is 0.71 (standard deviation 0.27). This larger relative variability in F1-score (coefficient of variation 38%) compared with accuracy (coefficient of variation 4.7%) provides quantitative support for our recommendation that F1-score and AUROC should be reported as primary metrics rather than accuracy. We strongly emphasize that establishing definitive superiority of any specific architecture or preprocessing strategy would require head-to-head comparison on a shared benchmark dataset, which does not yet exist in this field. This methodological gap is identified as a priority for future work in Section 4.13.

### 4.12. Clinical Impact: Workload Reduction, Diagnostic Agreement, and Real-World Implementation

Beyond raw classification performance, the clinical value of AI-assisted PT interpretation depends on tangible benefits to dermatological workflow and diagnostic reliability. The reviewed studies provide limited but instructive evidence on these dimensions. Regarding workload reduction, only Kim et al. [18] explicitly addressed this aspect by demonstrating that the YOLOv5x detection pipeline eliminates the need for manual cropping of individual allergen sites, a step that consumed an estimated 5–10 min per patient in their workflow. Extrapolated to a typical dermatology clinic performing 10–20 patch tests per week, automated detection could reduce technical reading time by approximately 1–3 h weekly. The classification-based approaches (Vezakis, Chan, Hall, Ravishankar) require manual site identification and therefore offer more limited workflow gains, primarily reducing subjective grading variability rather than overall reading time.

Regarding diagnostic agreement, Carter et al. [24] provides the only direct comparison between AI and human experts in a prospective setting. The AI model and board-certified dermatologists achieved comparable specificity (93% vs. expert reading), but expert readers demonstrated higher sensitivity than the model (58% AI sensitivity vs. higher human values). This pattern suggests that current AI models may function more reliably as a screening tool to confirm clearly negative reactions rather than as a definitive grading system. Inter-rater agreement metrics (Cohen’s kappa) were not reported in any of the reviewed studies, representing an important gap; future studies should report kappa statistics or weighted kappa for ordinal ICDRG grading to enable direct comparison with established inter-dermatologist agreement benchmarks (typically kappa = 0.4–0.6 for PT interpretation).

Regarding real-world implementation feasibility, several practical barriers remain insufficiently addressed in the current literature. None of the reviewed studies have progressed to regulatory approval (FDA, CE marking, or equivalent) as a medical device, and none have been integrated into commercial electronic health record (EHR) or dermatology image management systems. Computational requirements vary substantially: YOLOv5x and Xception require GPU inference for clinically acceptable latency, while smaller CNN architectures (Hall et al., Ravishankar et al.) can run on standard clinical computing hardware. Image acquisition standardization—including consistent camera positioning, lighting, and color calibration—represents the most significant practical barrier to widespread deployment, as performance demonstrated under controlled research conditions does not necessarily translate to routine clinical photography. Future implementation studies should report time-motion analyses, clinician acceptance metrics, and prospective comparisons of AI-assisted versus standard workflow in terms of reading time, diagnostic accuracy, and patient throughput.

### 4.13. Lessons Learned and Practical Recommendations

Based on the analysis of the reviewed studies, several key lessons and recommendations can be synthesized for researchers and clinicians interested in developing or implementing AI-based PT interpretation systems. First, regarding dataset construction, larger and more balanced datasets are essential. Prospective multi-center data collection efforts that specifically oversample rare reaction types (ICDRG grades ++ and +++) are strongly recommended. Second, regarding model selection, object detection frameworks such as YOLO offer practical advantages for clinical workflow integration by eliminating manual cropping, although they require more extensive annotation. For proof-of-concept studies with limited data, transfer learning with established CNN architectures such as EfficientNet remains a viable approach. Third, regarding evaluation methodology, studies must report balanced metrics including per-class sensitivity, F1-score, and AUROC rather than relying solely on overall accuracy. Fourth, regarding imaging standardization, studies should clearly document imaging equipment, lighting conditions, camera distance, and image resolution to enable reproducibility. When using smartphone or consumer cameras, color normalization and brightness standardization should be considered mandatory preprocessing steps. Fifth, regarding clinical validation, prospective external validation across multiple institutions and diverse patient populations should be the standard for studies claiming clinical applicability. Finally, regarding regulatory considerations, any AI system intended for clinical use in PT interpretation must meet applicable regulatory requirements for medical devices and demonstrate transparent, interpretable decision-making processes. For clinicians considering AI-assisted PT interpretation, the current evidence suggests that these tools should be viewed as supplementary aids that may improve consistency in routine screening, but all clinically significant findings should be confirmed by experienced dermatologists. Clinicians should request information about the training data demographics, validation methodology, and failure modes of any AI system before clinical adoption. For computer vision researchers entering this domain, the critical technical challenges include: (a) fine-grained discrimination between subtle reaction grades that differ primarily in erythema intensity and infiltration depth; (b) developing robust models that generalize across imaging devices, lighting conditions, and skin phototypes; (c) designing architectures that can integrate temporal multi-timepoint information; and (d) establishing standardized benchmark datasets and evaluation protocols to enable meaningful comparison across studies.

## 5. Conclusions

Artificial intelligence-based methods show potential as supportive tools for automated interpretation of patch test reactions. Deep learning models can identify clinically relevant reaction features and may improve the consistency and efficiency of patch test evaluation. However, existing evidence is constrained by small datasets, heterogeneous methodologies, and limited standardization. Notably, many studies employ binary classification (positive vs. negative), while the recognition of severe reactions according to the full ICDRG grading scale remains challenging. The analysis presented in this review suggests that object detection frameworks may offer practical advantages for clinical workflow integration, while comprehensive image preprocessing, particularly color normalization and brightness standardization, appears beneficial when imaging conditions are not strictly controlled. Performance evaluation should move beyond overall accuracy to include balanced metrics such as per-class sensitivity, F1-score, and AUROC, especially given the pronounced class imbalance inherent to PT datasets. These limitations indicate that current AI systems should be regarded as supplementary analytical instruments rather than fully autonomous diagnostic solutions. Considerable variability in imaging equipment, annotation practices, and preprocessing approaches further restricts direct comparison between studies and hinders the development of robust, reproducible methodologies. The absence of transformer-based approaches in the current literature represents an important gap, as these architectures have demonstrated competitive performance in related dermatological image analysis tasks. Strengthening methodological consistency, expanding dataset diversity, and pursuing multi-center external validation will be essential for ensuring that AI-assisted PT interpretation can be integrated responsibly and effectively into dermatological practice. Future work should focus on larger and more diverse datasets enriched with severe reaction types, harmonized image acquisition and preprocessing pipelines, transparent annotation workflows with quality oversight, and rigorous external validation across diverse patient populations to enable reliable clinical implementation. This review is subject to several limitations that should be acknowledged. First, the search was restricted to English-language publications, which may have excluded relevant studies published in other languages. Second, grey literature, conference abstracts without full-text availability, and preprints were excluded, potentially omitting emerging work not yet published in peer-reviewed journals. Third, the limited number of included studies (six) reflects the novelty of the field rather than a restrictive search strategy; however, this small sample size limits the strength of the conclusions that can be drawn. Fourth, the heterogeneity of study designs, datasets, and reported metrics precluded formal meta-analysis. Finally, this review was conducted by a limited number of reviewers, and no formal protocol was pre-registered, which may introduce selection bias despite adherence to PRISMA guidelines. Despite these constraints, this review fulfills an important role in the current research landscape. Independent bibliometric analyses and recent reviews [26,27,28] confirm that only six to eight original studies worldwide have applied deep learning specifically to PT image classification as of 2025, indicating that the present work captures the near-entirety of the available evidence base. By systematically synthesizing this emerging literature and identifying critical gaps in methodology, dataset construction, and clinical validation, this review provides a necessary foundation for future research efforts and establishes a benchmark against which subsequent progress in this field can be measured.

## Figures and Tables

**Table 1:** Summary of the studies included in this literature analysis on artificial intelligence-based automated patch test interpretation.

Author, Year	Study Type	No. of Patients	Dataset Size (Images)	Patch Test Protocol	Imaging Method	AI Architecture	Task	Performance Metrics
Kim S. et al., 2025 [18]	Prospective	3203	86,477	European baseline series, 30 allergens; evaluation at 24 h and 48 h; ICDRG 0–4 scale	DSLR photography	YOLOv5x	Object detection + multiclass classification	Accuracy: 98.3% F1-score: 0.982 Sensitivity: 0.997
Vezakis I.A. et al., 2023 [19]	Prospective	200	1579	European baseline series, 30 allergens; evaluation at 48 h and 72 h; ICDRG 0–4 scale	Antera 3D^®^ imaging system	EfficientNetB0 (CNN)	Binary classification	Accuracy: 90% F1-score: 0.83 Specificity: 94%
Chan W.H. et al., 2021 [20]	Prospective	77	5899	ACDS baseline series, 80 allergens; evaluation at day 4; ICDRG scale	Smartphone photography	Xception (CNN)	Binary classification	Accuracy: 99.5% F1-score: 0.89
Hall M.R. et al., 2024 [21]	Retrospective	201	2810 image tiles	Mayo Clinic baseline series, 80 allergens; evaluation at 24 h and 72 h; ICDRG scale	DSLR and smartphone photography	Custom 5-layer (CNN)	Object detection	Accuracy: 90.9% F1-score: 0.37 Specificity 91.7%
Ravishankar A. et al., 2024 [23]	Retrospective	125	13,622	Standard baseline series; evaluation at day 2 and day 4; ICDRG scale	DSLR and smartphone photography	CNN (binary classifier)	Binary classification	Accuracy: 90.1% AUROC: 0.940
Carter R. E. et al., 2024 [24]	Prospective	206	2060 sites	10 allergens per participant; evaluation at day 5; ICDRG scale; all Fitzpatrick types I–VI	Photograph (not specified)	Deep learning (CNN, ResNet-based)	Binary classification (prospective validation)	AUROC: 0.86 Sensitivity: 58% Specificity: 93%

AI—artificial intelligence; CNN—convolutional neural network; ICDRG—International Contact Dermatitis Research Group.

## Data Availability

No new data were created or analyzed in this study.

## Abbreviations

The following abbreviations are used in this manuscript:

AI	Artificial intelligence
DNNs	Deep learning networks
CNN	Convolutional neural network
PT	Patch test
ACD	Allergic contact dermatitis
ICDRG	International Contact Dermatitis Research Group
